# Utilizing Electromyographic Video Games Controllers to Improve Outcomes for Prosthesis Users

**DOI:** 10.1007/s10484-023-09598-y

**Published:** 2023-08-01

**Authors:** Shea McLinden, Peter Smith, Matt Dombrowski, Calvin MacDonald, Devon Lynn, Katherine Tran, Kelsey Robinson, Dominique Courbin, John Sparkman, Albert Manero

**Affiliations:** Limbitless Solutions, University of Central Florida, 12703 Research Pkwy Suite 100, Orlando, FL 32826 USA

**Keywords:** Surface electromyography, Serious gaming, Prosthetic, Upper limb, Biofeedback

## Abstract

A study was developed for a limb-different accessible video game controller that utilizes an electromyographic sensor to control gameplay actions. Data was collected from 50 college-aged student participants. This biofeedback-based serious game trains users in a virtual capacity, through the visualization of muscle contraction, via the movement of the video game character. The training platform has been developed to accompany the corresponding electromyographic actuated prosthetic arm device, leveraging the same control scheme to enable the translation of hand gesture states. This study evaluated the controller, user interface, and gameplay to identify training improvement outcomes and user satisfaction. Study participants were divided into two cohorts that differed in their intervention between the pre-test and post-test challenge course. Cohort one had a free play environment that encouraged learning through algorithmically generated track patterns and the use of powerups. In contrast, cohort two repeated the challenge mode, which was made up of a course of rings to jump through and focused on targeted muscle discretization via character jump heights correlated to muscle output. Data were collected to develop and validate training methods and identify overall game satisfaction and usability. The results of this study indicated an increase in the user’s ability to be successful based on time on task with the intervention. The study also evaluated the usability and participant experience with the intervention.

## Introduction

Prosthetic users experience many obstacles leading up to receiving a prosthesis and during their time learning to use their new device. Training and therapy are often cited as beneficial to reducing prosthetic rejection rates because they can aid in increasing the comfortability of the device; which is a prominent reason for rejection by new users (Wagner et al., 2007). Active training when receiving the device is also key in reducing upper limb prosthesis rejection, especially in pediatric populations (Davids, 2006). To supplement these benefits, the use of serious gaming to train prosthetic users is an additional area being explored. Serious gaming offers the benefit of continued and more frequent training sessions in addition to the scheduled time in therapy and with other providers on the user’s care team. This combination of training therapies offers the potential for a reduction in rejection rates of the device. The history of serious gaming shows its widespread usage and benefits to learning in healthcare, education, and psychology (Fleming et al., 2014; Gentry et al., 2019; Harris et al., 2008).

Serious gaming, is broadly defined as the utilization of gameplay to correspond with an intended goal (Gentry et al., 2019), such as learning how to control a prosthetic device by training certain transferable skills in the game. The proposed serious game utilizes an electromyographic controller and sensors that quantitatively measures muscle activity to correspond with the character’s jump heights through obstacles in an endless runner environment. Previous studies have used an EMG (electromyography) controller, the same input used for the prosthetic device, in addition to keyboard controls to play a video game (Dombrowski et al., 2017; Manero, 2018; Smith, 2022). This allows for learning the input controls for the prosthetic device while maintaining the familiarity that comes with keyboard controls (Dombrowski et al., 2017). However, the ideal gameplay and controller interaction design should be created to promote transferability between the tasks (Dombrowski et al., 2016; Manero, 2019). Similar studies have used an EMG controller to play a computer-based serious game without the need for additional controls (Khan et al., 2019). Creating a game using just the input from the prosthetic controller would allow attention to be focused on only learning one control schema (Dombrowski et al., 2016).

Challenges and obstacles in gaming have been shown to trigger learning and problem-solving skills in game-based learning situations (Fullerton, 2014). Literature on the subject has also shown that player mindset impacts how they approach obstacles; for example, gamers with a growth mindset do not allow mistakes to affect their attention to the game and thus perform better than fixed mindset players (Lee et al., 2012). Additionally, another study demonstrated increased interest in gaming due to the perceived benefit to their therapeutic plan (Prahm et al., 2017).

Biofeedback is a training technique that commonly uses surface electromyography to monitor the physiological functions occurring within the body and convert them into meaningful auditory or visual cues (Frank et al., 2010). Using biofeedback in a serious game allows users to visualize how their physical actions correlate to in-game performance to train muscle activity (Jitaree et al., 2012). The real-time visualization of muscle activity can allow behavior modification to correlate to the desired function output (Fernando & Basmajian, 1978; Wolf, 1983). Previous training studies have supported the rehabilitation of stroke patients, which showed clinically significant results with biofeedback, but was not necessarily a direct effect of biofeedback alone (Bradley et al., 1998). However, other research has discussed how biofeedback can be beneficial. A placebo-controlled study for hand rehabilitation that utilized biofeedback in conjunction with physical therapy saw positive results for involved patients (Armagan et al., 2003). Training to use a biofeedback-based training game for prosthetics and in-person therapy appointments may increase satisfaction and training outcomes with their device.

This study looked to determine if a correlation was present between time spent using the game and better score outcomes due to biofeedback-mediated gameplay. This study explored the impact of time on task across free-play and fixed path challenges. In both modes, the players were presented with an endless runner style game, where a character runs down a path and must jump to avoid obstacles. Each muscle contraction was measured by the electromyographic controller producing an analog signal to interpret. The signal was then discretized with user’s calibration of an applicable intensity range, and then converted in-game into the character’s jump height. A small ring below the character’s feet visualized the measured intensity, providing near real-time feedback for the participant between the player’s intent and the measured outcome. In the free-play modality, players were presented with an algorithmically generated set of jump challenges with no time limits. Player speed was increased as time progressed in the run. The game ends when the player runs into too many obstacles, raising the “mayhem” bar and subsequently ending the run. In the fixed ring challenge path, the player was presented with a pre-set, unchanging, challenge course set of hoops of varying height to jump through. While the time spent making the jumps is the same between both groups, the fixed path is used for both the pre and post-test. Cohorts were split so that one group would train via free-play while the other trained on direct ring challenge practice. In an optimized training game, practice would lead to improved performance, thereby allowing the game designers to come up with more interesting and varied play experiences maintaining a similar level of training.

## Methods

**Fig. 1:**
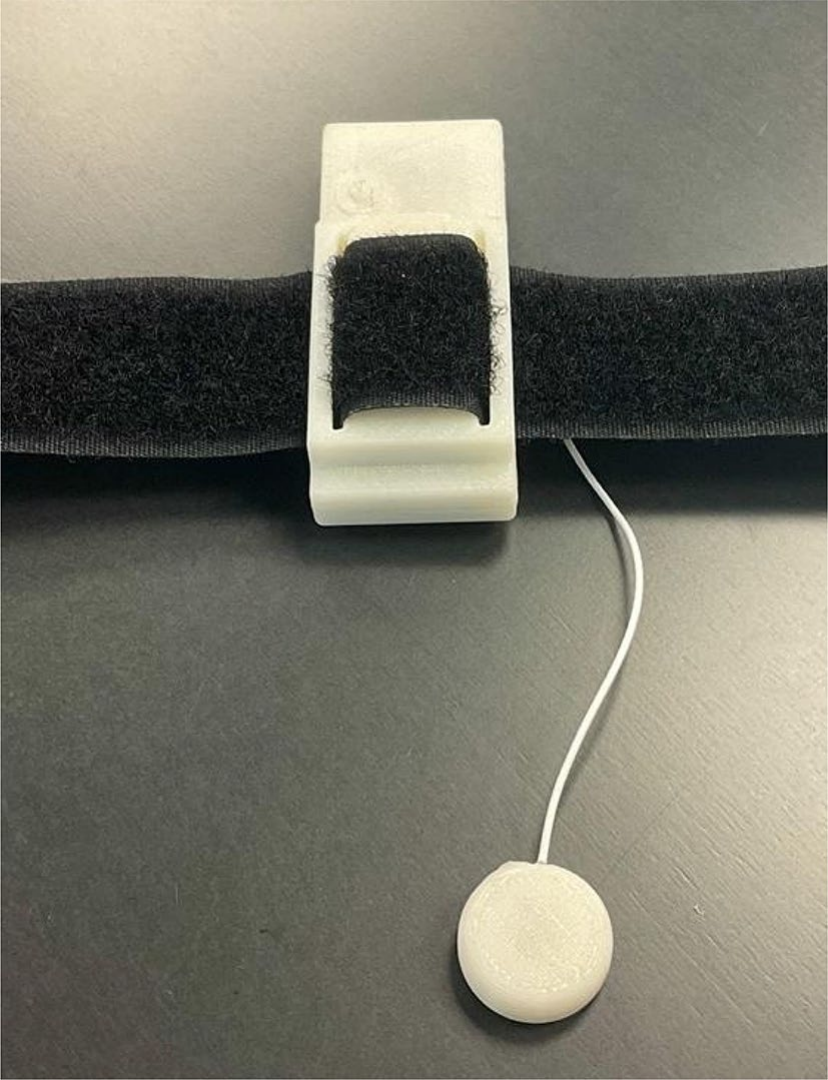
Novel bluetooth EMG video game controller

The authors’ research focuses on the development of 3D printed myoelectric bionic arm prosthetics, with incorporated artistic expression for children. The research facility and program are organized as Limbitless Solutions at the University of Central Florida. To improve pediatric patients’ experiences with the prosthetic device, a serious video game was designed to train the muscle isolation and contractions required to control the electromyography-actuated prosthetic. In order to evaluate the usability and efficacy of the video game, students at the University of Central Florida with complete muscular control of their upper extremities were asked to participate in play testing and evaluation of the training platform. To train muscle flexion in the upper limb, a novel Bluetooth EMG video game controller was employed. An image of the controller is shown in Fig. 1*.* The controller utilizes the signal received by EMG sensors placed on the brachioradialis or biceps brachii (long) muscle group on the preferred side of the participant. The EMG controller is paired via Bluetooth to the game *Limbitless Runner*: an endless runner style of game. Based on the cohort each participant was selected for, users participated in a ring challenge mode which encouraged jumping at different heights of their character. The heigh of the character’s jump was dictated by the relative strength of contraction of the muscle, having been calibrated before the start of play. The free play environment encouraged users to explore character motions through in-game incentives. Incentives included stars and powerups that users could collect and gave more freedom in flexing and movement of the character as opposed to the more focused muscle discretization that the challenge mode targeted.

**Fig. 2:**
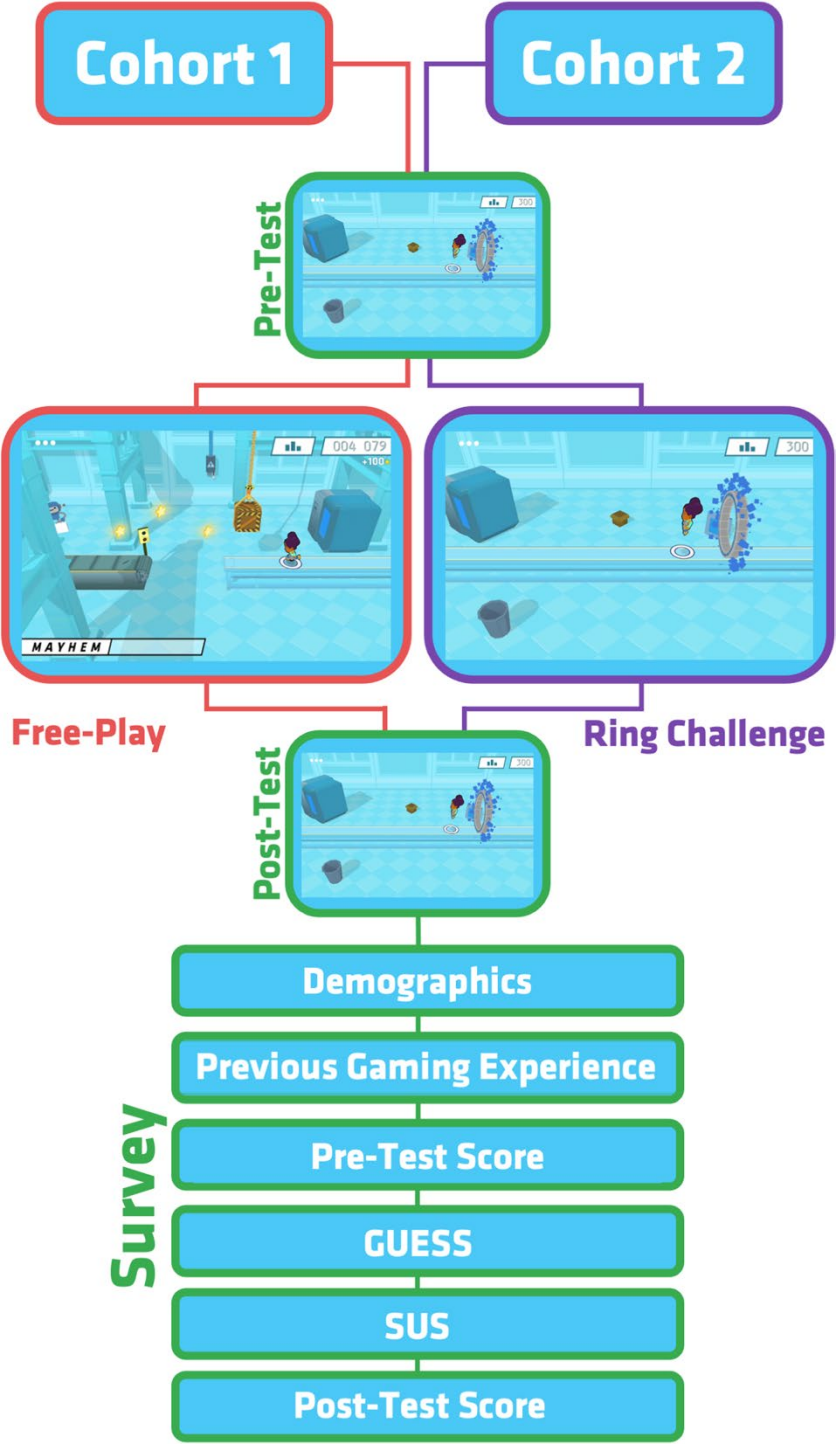
Methodology for cohort 1 and cohort 2 for study intervention and data collection

This study divided participants into two cohorts to test gameplay improvement. A visualization of each cohort’s evaluation methods is presented in Fig. 2. Cohort one completed their pretest in the ring challenge mode, followed by 15 min in the free-play game mode before taking the post-test in the ring challenge mode. Cohort two followed a similar testing route, with a pretest in the challenge mode followed by three rounds of the ring challenge mode before moving on to the post-test in the ring challenge mode. Participants were given breaks as necessary and a mandatory 5-min break to reduce the influence of muscle fatigue before their final run. Both cohorts then completed surveys via the Qualtrics surveying platform (Qualtrics, Provo, UT): an American based company that develops software that assists in collection and organization of feedback and data. Surveys were designed to collect data for demographics, previous gaming experience, pre-test scores, game user experience satisfaction (GUESS) (Phan et al., 2016), system usability (SUS) (Brooke, 1996), and finally, their post-test score data. The Game User Satisfaction Survey (GUESS) is a validated gaming scale used as a comparative measure of user satisfaction with their gaming experience (Phan et al., 2016). GUESS is a 7-point Likert scale used to measure the general likeability of the game. A score of 4 points represents “neither agree nor disagree”, with higher values corresponding to stronger agreements to the statements. The SUS is a standardized measure to define a reference point for usability by covering the game’s effectiveness, efficiency, and satisfaction (Brooke, 1996).

## Results

**Fig. 3:**
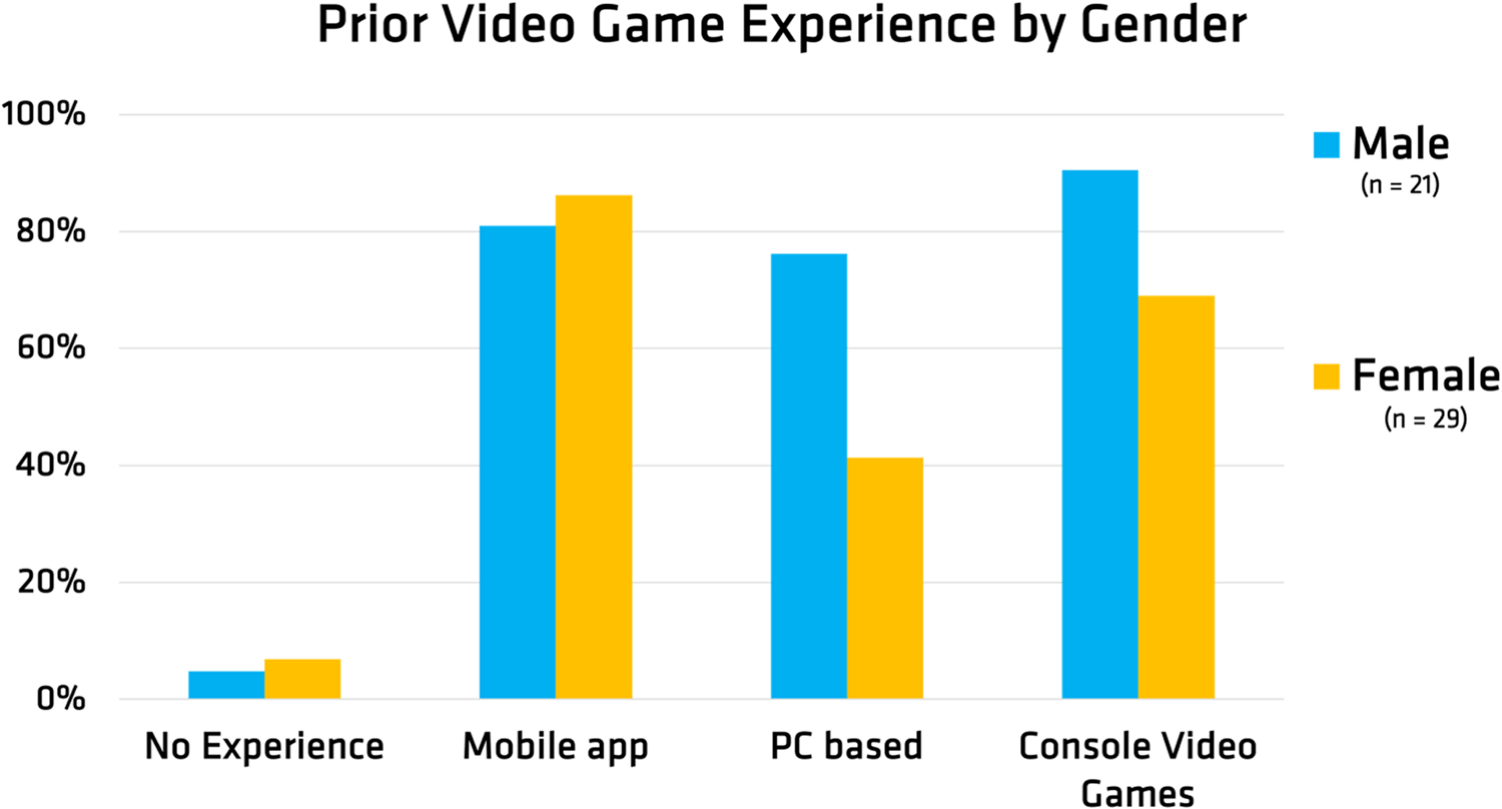
Prior video game experience reported normalized by reported gender (female n = 29, male n = 21)

**Fig. 4:**
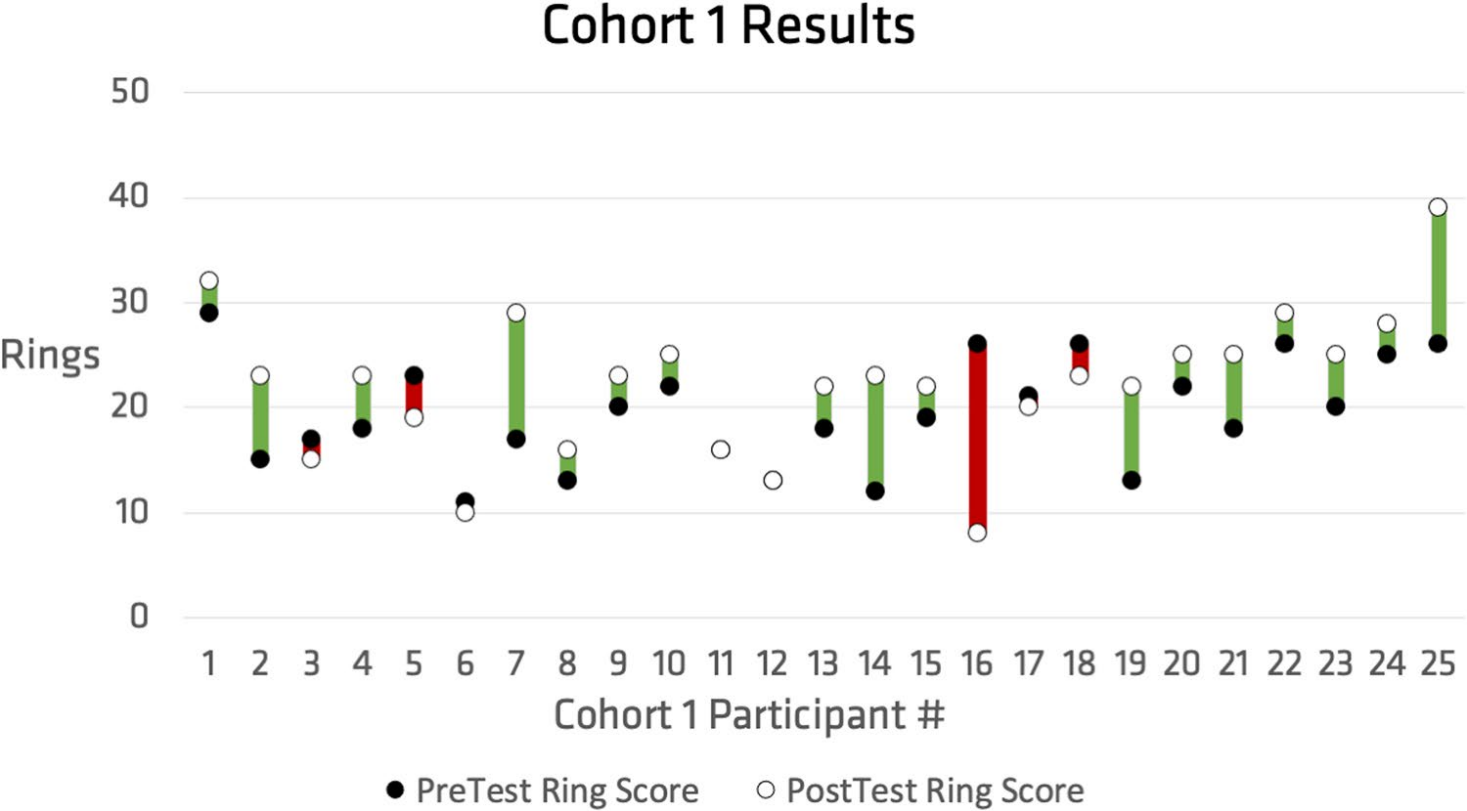
Comparing the pre (filled point) and post (open point) ring test challenge for cohort 1, who participated in the free-play intervention, with increasing scores tracked in green and decreased scores tracked in red (Color figure online)

**Fig. 5:**
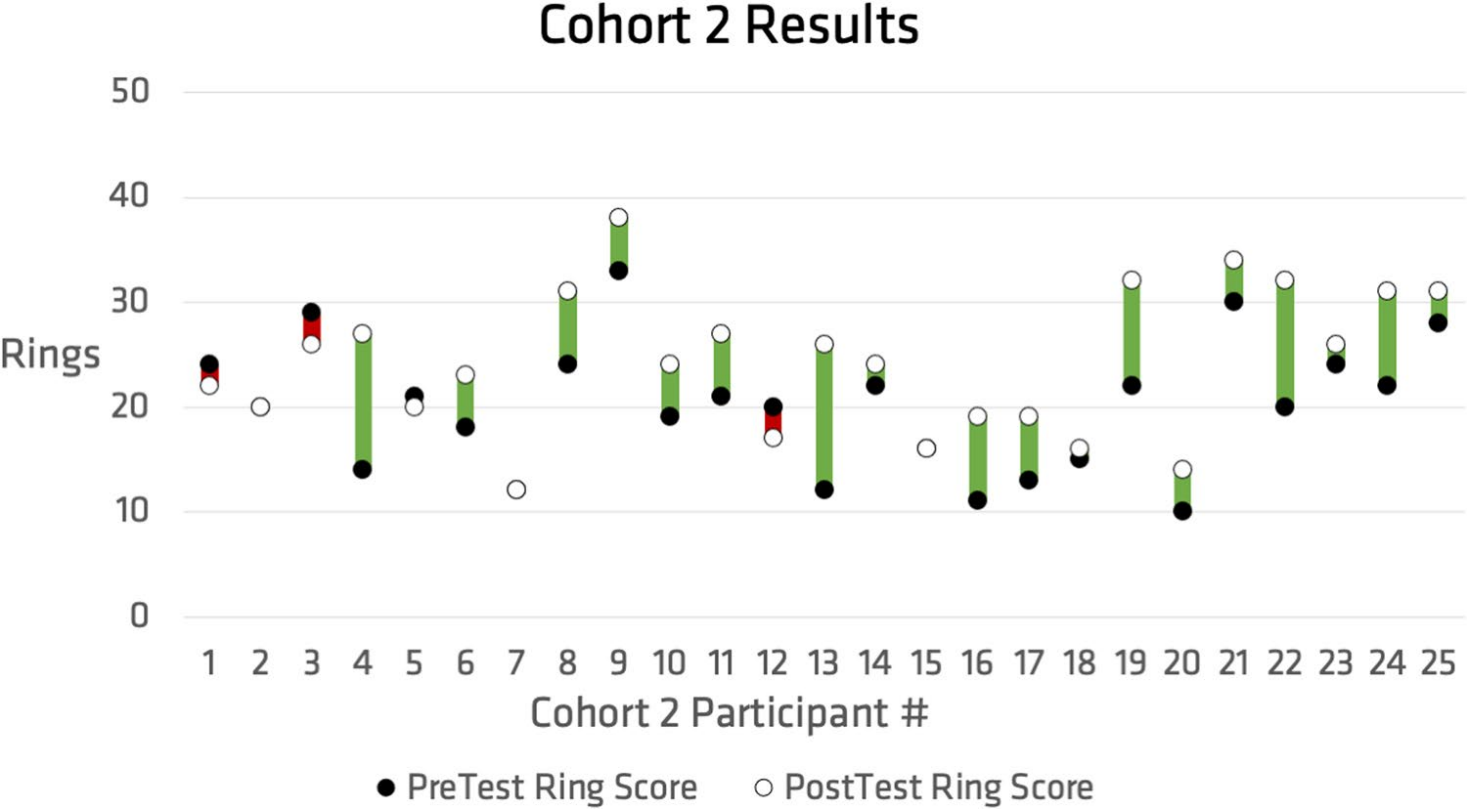
Comparing the pre (filled point) and post (open point) ring test challenge for cohort 2, who participated in the on-task ring challenge intervention, with increasing scores tracked in green and decreased scores tracked in red (Color figure online)

Demographic information was collected with a focus on prior experience with video games. The study collected data with twenty-one male participants and twenty-nine female participants, for a total sample size of fifty participants. Prior experience with video games, normalized for each demographic, is presented in Fig. 3. Results of the ring challenge mode revealed that regardless of which cohort participants were in, scores were generally increased from the pretest to the post-test. The scoring for each cohort, pre and post, are presented in Figs. 4 and 5. These results support the original hypothesis that time on task with the game and the novel control device would improve score outcomes, which indicates the ability to discretize muscle contraction for controlling the multi-gesture bionic arm.

Each cohort had twenty-five participants in total. Seventeen participants in cohort 1 showed improvement in their post-test score compared to their pretest score. Eighteen participants in cohort 2 showed improvement in their post-test scores compared to their pre-test scores. Thus, out of the fifty total participants, thirty-five showed improvements from their pre-test to their post-test.

**Fig. 6:**
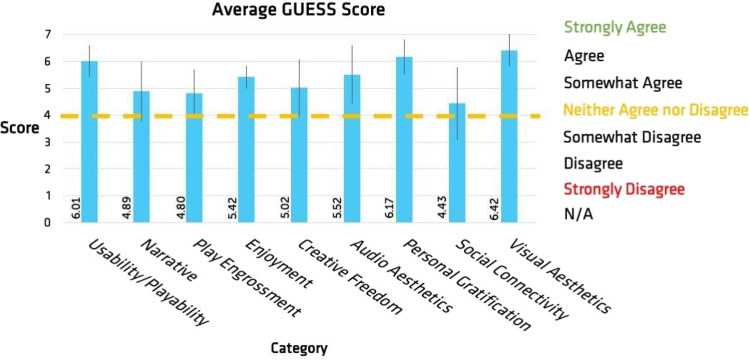
Comparing the average Game User Experience Satisfaction Survey (GUESS) for each of the 9 categories including Usability/Playability, Narrative, Play Engrossment, Enjoyment, Creative Freedom, Audio Aesthetics, Personal Gratification, Social Connectivity and Visual Aesthetics

The results for all participants’ GUESS surveys are presented in Fig. 6. GUESS categories include usability/playability, narratives, player engrossment, creative freedom, audio aesthetics, personal gratification, social connectivity, and visual aesthetics (Phan et al., 2016). The categories received average values of 6.01, 4.89, 4.80, 5.42, 5.02, 5.52, 6.17, 4.43, and 6.42, respectively. The data points to the visual aesthetics, personal gratification, and usability/playability aspects of the game were found to have the strongest positive influence on how the end user viewed their satisfaction with their gaming experience.

## Game User Experience Satisfaction Survey (GUESS)

Upon analysis of survey results, the section of the survey measuring System Usability Survey (SUS) indicated that the overall participant scoring of the system was 81.70 on a 100-point scale, collected from the fifty participant’s responses. Jeff Sauro and Jim Lewis developed a measure of the System Usability Survey, which gives context to the grade 81.70 as scoring in the 90–95% percentile quality range of systems graded by SUS (Bangor et al., 2008, 2009; Sauro, 2012, 2018). Acceptable scores on the SUS scale are above 70, while below 50 is considered unacceptable (Bangor et al., 2008, 2009; Sauro, 2012, 2018).

## Discussion

This study evaluated a novel Bluetooth EMG controller and associated serious game’s user interface and gameplay to identify training improvement outcomes and user satisfaction. Perceptions of the controller’s usability were overall positive, rating the controller as an acceptable control interface for the video game training. Regarding the therapeutic outcomes of the video game: participants performed significantly better on the post assessments following video game training, irrespective of cohort. This is believed to be due to the positively perceived controller’s usability and its efficacy in conjunction with the serious game. This provides positive evidence for future use of the video game training and its potential benefit in future clinical trials, which will be further expounded on later in this section.

Participants in Limbitless’ bionic arm-related clinical trials are provided multiple EMG video games as a supplemental training method to aid in learning how to utilize their prosthetic. The initial state of the arm includes only open and close functionality, gross grasp, mapped to any decipherable muscle contraction by the EMG sensor above a noise threshold. As the participants in these trials play the games, they learn to contract their muscles in a repeatable and discretized way. Using a single EMG sensor, multiple inputs can be discretized and assigned a different hand state gesture. With sufficient experience, patients will level up in the games and can be rewarded by unlocking additional multi-gesture support in their bionic arm. This stair-stepping approach ensures that the initial usability of the prosthetic minimizes frustration, while training with the gamified simulation can provide a lower stress environment to practice muscle isolation and discretization.

Even the most engaging games can have users lose interest if the challenges are repeated for a long duration. This endless runner style game provides an algorithmically generated track, generating less repetitive gameplay with unique interactions in the free play mode. The fixed challenge course described in the methods section provides a second gameplay mode that is more focused on achieving specified jump heights from muscular discretization. This study looked at the impact of free play versus a fixed challenge for participants. Data collected identified both modes of training as having a positive effect on the post test, ring challenge mode, scoring.

The initial investigation looked to evaluate the gameplay abilities resulting from training in both free play mode and the more structured challenge mode. Participants were divided into cohorts 1 and 2, which differed in their tasks between pre and post-test. Different cohorts were used to investigate the impact of their gameplay experience on their training. Cohort 2 was observed to have a greater average increase in score (4.28) than cohort 1 (2.76). However, based on the data presented above, a statistically significant link (p < α = 0.05) was not able to be identified between cohort design and score improvement (p = 0.17). However, the results indicate that post-training scores significantly increased overall (p = 2.375 × 10 ^(−5)) irrespective of which cohort participants were a part of. This result points to increased time on task as beneficial to learning device controls. This indicates that while there is a statistically significant increase based on time on task, the free play mode does not significantly affect the training differently than the challenge mode. This is a positive outcome because both modes of training were deemed supportive of training outcomes and the diversity of gameplay challenges will enhance the game’s longevity.

The data collected using the on-campus population is asserted to be generalizable in specific contexts for the planned future patient group composed of pediatric limb different patients. Evaluation of the user interface, satisfaction, and their experience learning the system is anticipated to be generalizable from a predominantly university-aged student to a planned population of pediatric patients. This is anticipated due to the originality of the game controller device and that neither college-aged students nor pediatric children will have been exposed to this EMG video game control system. Both populations are also expected to experience similar learning experiences as their conditions for learning (CFL) and the environment in which they are learning are presumably identical (Cantor et al., 2019). The more matured population of university aged students was anticipated to provide more clear and nuanced feedback on usability for the device and software. They are also expected to provide efficient feedback on the game’s readiness for application during its early development phase, proving even more useful. This population is preferred so that future studies with vulnerable pediatric populations will be efficient in their proceedings and use of study interventions. Usability perceptions regarding the interface, menus, and calibration are anticipated to be applicable across the populations. Since both groups were unfamiliar with the control device, accurate calibration for proper muscle contraction feedback was necessary to gain the most significant learning benefits.

The free-play mode provides a randomized experience with a slowly increasing game speed, making the challenge rise while providing a unique experience every play through of the game. Applying Bartle’s player types of *Achievers, Explorers, Socializers*, and *Killers*, the two modes represent play structures that can satisfy players with both an achiever or explorer mentality (Bartle, 1996). The achievers may find more enjoyment from hitting a perfect score in the hoop challenge or unlocking new cosmetics for the player’s character. Explorers meanwhile may enjoy discovering new obstacles in the game or seeing a new background. While not explicitly researched here, Socializers may enjoy the high score tables. In these games, primarily designed for training kids, special care is made to reduce opportunities for Killer player types. Other games in the catalog provide multiple paths through the game and require multiple playthroughs to complete. These results indicate that the choice being provided in the games is not negatively impacting the training outcomes.

Initial study challenges include identifying optimal EMG placement and calibration for an optimized gaming experience utilizing serious gaming and biofeedback. The electrode placement has minor variation for each individual due to how they contract their muscles and prefer to engage the system. This is similar to placement for players in the limb difference community, except players can be coached to consider moving their hands to activate muscles in their arms which can accelerate comprehension. The novelty of the control still leads to an apparent learning curve in controlling the EMG input. Proper calibration will mitigate placement errors, as long as the participant is able to readily contract the muscle group the controller is placed on. Users of the actual prosthetic device might be limited in calibration and placement options based on their particular limb difference geometry and physiology and the prosthetic’s socket position at the skin interface.

This initial evaluation is important to identify early user interface design successes and design challenges, which can be used as a feedback loop for training design. Further studies will be conducted on using the device for users with limb differences to be evaluated in combination with prosthetic usage and mastery.

## Data Availability

The data collection surveys for this manuscript were generated using Qualtrics software, Version 2022 of Qualtrics. Copyright © 2023 Qualtrics. Qualtrics and all other Qualtrics product or service names are registered trademarks or trademarks of Qualtrics, Provo, UT, USA. https://www.qualtrics.com.
