# Correction to: Utilizing Electromyographic Video Games Controllers to Improve Outcomes for Prosthesis Users

**DOI:** 10.1007/s10484-024-09636-3

**Published:** 2024-03-10

**Authors:** Shea McLinden, Peter Smith, Matt Dombrowski, Calvin MacDonald, Devon Lynn, Katherine Tran, Kelsey Robinson, Dominique Courbin, John Sparkman, Albert Manero

**Affiliations:** Limbitless Solutions, University of Central Florida, 12703 Research Pkwy Suite 100, Orlando, FL 32826 USA

**Correction to: Applied Psychophysiology and Biofeedback (2023) 49:63–69**

10.1007/s10484-023-09598-y

In the original version of this article unfortunately missed to include the references for the three in-text citations - Manero et al., 2018, 2019; Smith et al., 2022.

The complete reference details are as follows:

Manero, A., Sparkman, J., Dombrowski, M., Buyssens, R., & Smith, P. A. (2018). Developing and Training Multi-gestural Prosthetic Arms. In J. Y. C. Chen & G. Fragomeni (Eds.), Virtual, Augmented and Mixed Reality: Interaction, Navigation, Visualization, Embodiment, and Simulation (Vol. 10,909, pp. 427–437). Springer International Publishing. 10.1007/978-3-319-91581-4_32.

Manero, A., Smith, P., Sparkman, J., Dombrowski, M., Courbin, D., Barclay, P., & Chi, A. (2019). Utilizing additive manufacturing and gamified virtual simulation in the design of neuroprosthetics to improve pediatric outcomes. MRS Communications, 9(3), 941–947. 10.1557/mrc.2019.99.

Smith, P., Dombrowski, M., McLinden, S., MacDonald, C., Lynn, D., Sparkman, J., Courbin, D., & Manero, A. (2022). Advancing Dignity for Adaptive Wheelchair Users via a Hybrid Eye Tracking and Electromyography Training Game. 2022 Symposium on Eye Tracking Research and Applications. 10.1145/3517031.3529612.

The original article has been corrected.

